# Targeting Autophagy and the Anticipatory Unfolded Protein Response Leads to Increased Breast Cancer Cell Death

**DOI:** 10.17912/micropub.biology.001376

**Published:** 2025-02-20

**Authors:** Jeffrey Brooks, Antonina Pizzo, Caela Fedraw, Florina Gojcaj, Myles Harris, Destiny Proffett, Ahed Anbari, Liselle Tungol, Mara R. Livezey

**Affiliations:** 1 Chemistry & Biochemistry, University of Detroit Mercy, Detroit, Michigan, United States

## Abstract

Activation of the anticipatory unfolded protein response (aUPR) by the small molecule 3,3-bis(4-hydroxyphenyl)-7-methyl-1,3,dihydro-2H-indol-2-one (BHPI) leads to necrotic cell death in a variety of cancer cells containing Estrogen Receptor alpha (ERα). A key feature of BHPI’s mechanism of action is depletion of cellular ATP. Other pathways such as autophagy can regulate cellular energy levels and ATP production. We present data that suggests targeting both the aUPR and autophagy leads to a significant increase in cell death in T47D and TYS breast cancer cells treated with BHPI. This combination presents itself as a possible therapeutic strategy against breast cancer.

**Figure 1. BHPI inhibits autophagy and further inhibition with chloroquine (CQ) increases BHPI’s efficacy.**
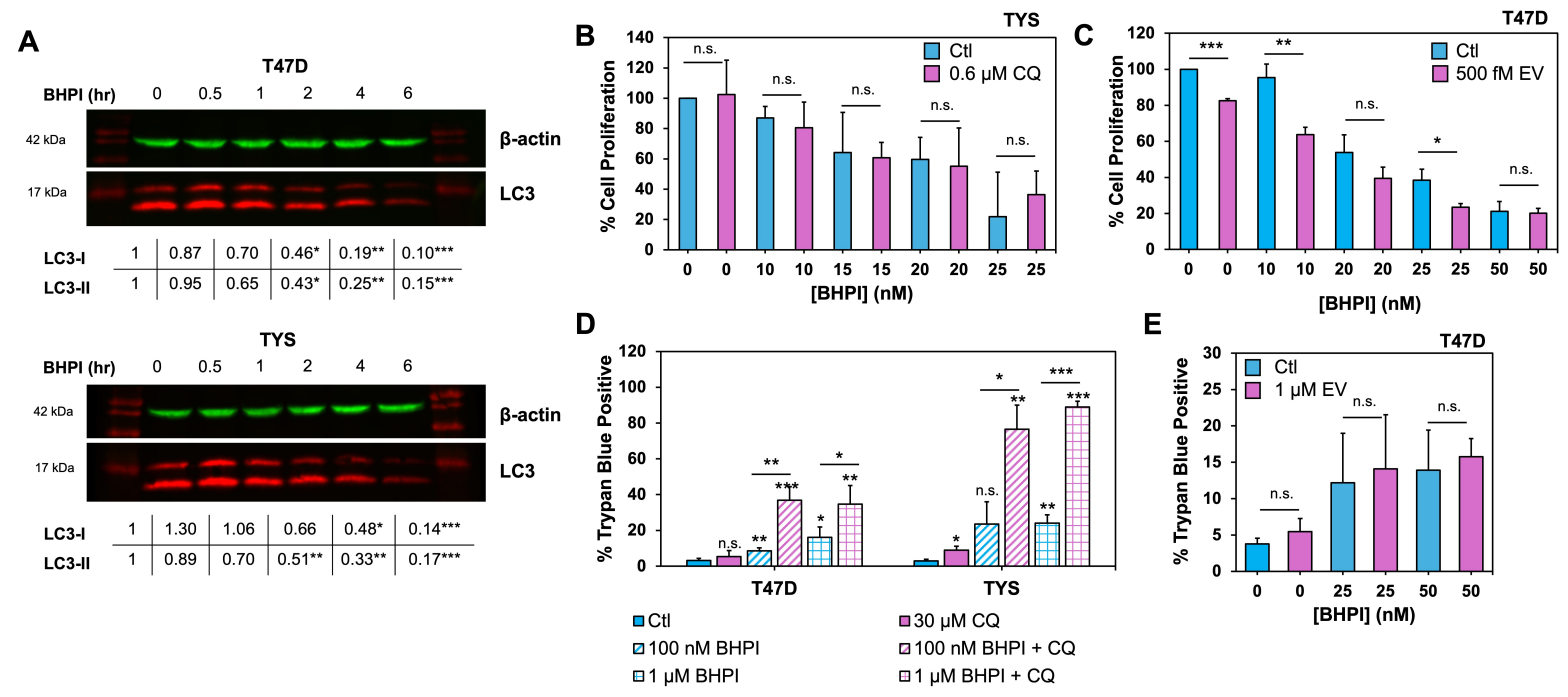
A) Representative western blot images of LC3 production in T47D and TYS cells. Cells were pre-treated with 30 µM CQ for 24 hours to stop autophagy turnover, then treated for the indicated time with 1 µM BHPI. Quantitation of LC3-I and LC3-II is below each blot, normalized to β-actin. B) TYS cell proliferation after 3 days treatment with DMSO, CQ, or BHPI. C) T47D cell proliferation after 3 days treatment with DMSO, everolimus (EV), or BHPI. D) Trypan blue exclusion assay of T47D and TYS cells treated with CQ and BHPI for 24 hours. E) Trypan blue exclusion assay of T47D cells treated with EV and BHPI for 24 hours. For A-E data are mean ± standard deviation of n = 3 biological replicates (in D, T47D cells are n = 4 biological replicates), *p<0.05, **p<0.01, ***p<0.001, n.s.= not significant by Student’s t-test.

## Description

Many anti-cancer drugs trigger apoptotic cell death, specifically caspase-dependent apoptosis (Koren & Fuchs, 2021; Peng et al., 2022) . However, many cancer types are resistant to apoptosis *de novo* or develop resistance, and caspase-3 is downregulated in many human breast cancers (Devarajan et al., 2002; Hanahan & Weinberg, 2011) . Thus, development of therapies that work differently is of interest. 3,3-bis(4-hydroxyphenyl)-7-methyl-1,3,dihydro-2H-indol-2-one (BHPI) is a small molecule biomodulator of Estrogen Receptor alpha (ERα) that leads to strong and sustained activation of the anticipatory unfolded protein response (aUPR). Under mild activation by molecules such as estrogen, the aUPR is a protective mechanism that promotes cell proliferation by regulation of intracellular protein homeostasis, however, this pathway becomes lethal when activated by BHPI (N. Andruska et al., 2015; N. D. Andruska et al., 2015; Livezey et al., 2018; Zheng et al., 2018). Key features of BHPI’s mechanism of action are calcium efflux from the endoplasmic reticulum, near-quantitative protein synthesis inhibition, cellular swelling, and ATP depletion (N. Andruska et al., 2015; N. D. Andruska et al., 2015; Livezey et al., 2018; Zheng et al., 2018). Cellular ATP depletion is the result of a futile cycle of calcium leaking from endoplasmic reticulum inositol 1,4,5-trisphosphate receptors (IP _3_ R), which is then pumped back in via ATP-dependent SERCA pumps, only to leak back through IP _3_ R channels that remain open due to BHPI-activated IP _3_ production. Recently, it was shown that calcium efflux from the endoplasmic reticulum also activates transient receptor potential melastatin member 4 (TRPM4), resulting in sodium intake from the extracellular space. Sodium intake is required for sustained aUPR activation, cell swelling, ATP depletion, and necrotic cell death (Ghosh et al., 2023; Livezey et al., 2018) .

BHPI’s mechanism of action is novel and results not only in inhibition of cell proliferation, but also cell death across many ERα positive breast, ovarian, and endometrial cell lines (Livezey et al., 2018) . However, BHPI is not potent enough to completely eradicate cells grown in 2-dimensional cell culture or mouse xenografts (N. D. Andruska et al., 2015; Livezey et al., 2018; Zheng et al., 2018). When used in combination with broadly toxic chemotherapeutic drugs such as Paclitaxel, BHPI is able to eradicate even multidrug resistant xenografts such as OVCAR-3 (Zheng et al., 2018) . It has not been explored whether BHPI’s efficacy would increase when used in combination with other less-toxic compounds. Furthermore, combination treatment with small molecules that might synergize with aspects of BHPI’s mechanism of action, such as depletion of cellular ATP, is also unexplored.

Autophagy is a cellular self-recycling mechanism that contributes to energy homeostasis. Its involvement in cancer is complex, in that it can be either protective or harmful to cancer (Deegan et al., 2014; Kaur & Debnath, 2015; Ogata et al., 2006) . Chloroquine (CQ) is an anti-malarial drug that blocks lysosomal acidification and therefore inhibits turnover of cellular components in autophagy. A number of studies have reported that treatment of cancer cells with CQ increases the cells’ sensitivity to a variety of treatments including cisplatin, 5-fluorouracil, paclitaxel, PI3K/AKT inhibitors, antiestrogens, and radiation therapy (Cocco et al., 2022; Cook et al., 2014; Ferreira et al., 2021; Xu et al., 2018) . Targeting autophagy in addition to the aUPR is of interest, since inhibition of autophagy may lead to further reduction in cellular ATP, and increased cell death. Conversely, activating autophagy with everolimus (EV) might rescue cells from BHPI. EV is an inhibitor of mammalian target of rapamycin (mTOR), that leads to activation of autophagy. We, therefore, explored the impact of activating and inhibiting autophagy with EV and CQ respectively, on BHPI’s efficacy against ERα positive breast cancer cells.

LC3 is a protein involved in the elongation phase of autophagy, as well as formation of the autophagosome (Sano & Reed, 2013) . In as little as 2 hours, BHPI caused a significant reduction in both LC3-I and LC3-II levels in T47D and TYS cells ( Figure 1A ). Since LC3-I conversion to LC3-II is important for the elongation phase of autophagy (Sano & Reed, 2013) , BHPI inhibits autophagy to some extent on its own in breast cancer cells. However, treatment of cells with BHPI leads to near-quantitative protein synthesis inhibition in many cell types including T47D and TYS (N. D. Andruska et al., 2015; Mao et al., 2016). Therefore, whether BHPI truly inhibits autophagy, or simply decreases LC3 levels through protein synthesis inhibition is unclear.

We next explored how inhibition or activation of autophagy would impact BHPI’s efficacy. To determine if further inhibition of autophagy with CQ leads to greater inhibition of cellular growth when used in combination with BHPI, 3-day proliferation experiments were performed. While BHPI alone causes a decrease in cell proliferation in TYS cells that are highly sensitive to BHPI, no further significant decrease was seen in cells co-treated with BHPI and CQ ( Figure 1B ). Furthermore, we utilized EV to activate autophagy. No substantial difference was seen in T47D cells treated with BHPI, or in combination with EV ( Figure 1C ). It is important to note that small concentrations of both CQ and EV were used in these experiments, as higher concentrations found in literature lead to significant inhibition of cell proliferation on their own. Therefore, 0.6 µM CQ and 500 fM EV were chosen after dose-curves were performed to determine concentrations that would not significantly inhibit proliferation of T47D and TYS cells.

Treatment of cells with BHPI results in significant cell death in as little as 1 hour in TYS cells, and within 24 hours in most ERα positive cell lines including T47D (Livezey et al., 2018) . Death was measured in T47D and TYS cells via trypan blue exclusion after 24 hours. Treatment of cells with 30 µM CQ in addition to 100 nM or 1 µM BHPI resulted in significantly more death in T47D and TYS cells, compared to treatment with BHPI alone ( Figure 1D ). At 1 µM BHPI in T47D cells and 100 nM BHPI in TYS cells, co-treatment with CQ increased cell death significantly, but modestly. The mixed-efficacy of co-treatment across concentrations and cell types requires further study, especially using additional models such as 3-dimensional cell culture or xenografts, to determine if the toxic effect is synergistic and replicable *in vivo* . 30 µM CQ was chosen as this concentration is largely non-toxic across a range of cell types (Morgan et al., 2014; Sharma et al., 2012; Yang et al., 2020) , but is sufficient to inhibit autophagy (Klionsky et al., 2021; Sharma et al., 2012) . Indeed, we saw minimal cell death induced by CQ alone ( Figure 1D ). However, treatment of T47D cells with EV did not rescue them from BHPI, even at low nM concentrations of BHPI ( Figure 1E ). It has previously been shown that BHPI has a threshold effect, where a change in concentration as small as from 10 nM to 25 nM can have a dramatic increase in efficacy ( Figure 1B,C ) (N. D. Andruska et al., 2015; Mao et al., 2016). Given this, we hypothesize that reversing BHPI is challenging once this threshold is reached, and that even at low nM concentrations of BHPI, EV is not sufficiently potent.

Combination treatment of ERα positive breast cancer cells with BHPI and CQ led to a significant increase in cell death compared to BHPI alone. Inhibition of autophagy further increases BHPI’s efficacy, however the reason why remains unexplored, though enhanced depletion of cellular ATP is likely. Also unexplored is whether the enhancing effect of CQ with BHPI is synergistic, and if this result is replicable in other ERα positive breast cancer cell lines or cancer models. Additionally, since BHPI seems to inhibit autophagy to some extent on its own, it is of interest to explore how activation of the aUPR by BHPI connects to autophagy. Our findings suggest that simultaneous targeting of the aUPR with BHPI and autophagy may be a viable therapeutic strategy for ERα positive breast cancer.

## Methods

*Reagents*

BHPI was obtained from Tocris Bioscience. Chloroquine, everolimus, resazurin sodium salt (Alamar Blue), Trypan Blue, MEM, and FBS were obtained from Sigma Aldrich.

*Cell Culture and Maintenance*

T47D human breast cancer cells, that contain wild type ERα, and TYS cells that contain the constitutively active ERαY537S mutation were obtained from D.J. Shapiro (Mao et al., 2016) . T47D cells are maintained in MEM with 10% fetal bovine serum (FBS), and TYS cells are maintained in MEM with 10% charcoal-dextran-FBS stripped of hormones. All experiments are performed before passage number 30.

*Cell Proliferation Assay*

T47D and TYS cells were plated at 4,000 cells/well in a 96-well plate. The next day, media was changed to that containing the indicated treatment. Cells were grown for 3 days and measured with Alamar Blue on the third day. Control cells treated with DMSO vehicle were set to 100% proliferation.

*Trypan Blue Exclusion Assay*

T47D and TYS cells were plated at 250,000 cells/well in a 6-well plate. The next day, media was changed, and the indicated treatments were added. 24 hours after treatment, cells were harvested and concentrated to 2-5 million cells/mL, mixed with trypan blue, and immediately read on a Countess-II FL automatic cell counter (ThermoFischer).

*Western Blot*

T47D and TYS cells were pre-treated with 30 µM CQ for 24 hours. 1 µM BHPI was then added for the indicated time before cells were harvested in RIPA buffer (N. Andruska et al., 2012, 2015). Western blots were performed using LC3A/B (Cell Signaling Technology, # 4108) and β-actin (Sigma, A1978) primary antibodies, with secondary antibodies from Li-COR (926-68071, 926-32210). Images were taken on a Li-COR Odyssey Fc in Image Studio and were analyzed in Empiria Studio, with β-actin as the loading control.
